# A program to respond to otitis media in remote Australian Aboriginal communities: a qualitative investigation of parent perspectives

**DOI:** 10.1186/s12887-018-1081-3

**Published:** 2018-03-06

**Authors:** Caroline Jones, Mridula Sharma, Samantha Harkus, Catherine McMahon, Mele Taumoepeau, Katherine Demuth, Karen Mattock, Lee Rosas, Raelene Wing, Sulabha Pawar, Anne Hampshire

**Affiliations:** 1 MARCS Institute, ARC Centre of Excellence for the Dynamics of Language, Western Sydney University, Locked Bag 1797, Penrith, NSW 2751 Australia; 2 ARC Centre of Excellence for Cognition and its Disorders, Macquarie University, Sydney, Australia; 3 Audiology Program, Department of Linguistics, Macquarie University, HEARing CRC, Sydney, Australia; 4 Australian Hearing, Sydney, Australia; 5 Department of Psychology, University of Otago, Dunedin, New Zealand; 6 Department of Linguistics, Macquarie University, Sydney, Australia; 7 Sunrise Health Service, Katherine, Northern Territory Australia; 8 The Smith Family, Sydney, Australia

**Keywords:** Hearing loss, Indigenous, Interventions, Otitis media, Qualitative

## Abstract

**Background:**

Indigenous infants and children in Australia, especially in remote communities, experience early and chronic otitis media (OM) which is difficult to treat and has lifelong impacts in health and education. The LiTTLe Program (Learning to Talk, Talking to Learn) aimed to increase infants’ access to spoken language input, teach parents to manage health and hearing problems, and support children’s school readiness. This paper aimed to explore caregivers’ views about this inclusive, parent-implemented early childhood program for 0–3 years in an Aboriginal community health context.

**Methods:**

Data from in-depth, semi-structured interviews with 9 caregivers of 12 children who had participated in the program from one remote Aboriginal community in the Northern Territory are presented. Data were analysed thematically. Caregivers provided overall views on the program. In addition, three key areas of focus in the program are also presented here: speech and language, hearing health, and school readiness.

**Results:**

Caregivers were positive about the interactive speech and language strategies in the program, except for some strategies which some parents found alien or difficult: such as talking slowly, following along with the child’s topic, using parallel talk, or baby talk. Children’s hearing was considered by caregivers to be important for understanding people, enjoying music, and detecting environmental sounds including signs of danger. Caregivers provided perspectives on the utility of sign language and its benefits for communicating with infants and young children with hearing loss, and the difficulty of getting young community children to wear a conventional hearing aid. Caregivers were strongly of the opinion that the program had helped prepare children for school through familiarising their child with early literacy activities and resources, as well as school routines. But caregivers differed as to whether they thought the program should have been located at the school itself.

**Conclusions:**

The caregivers generally reported positive views about the LiTTLe Program, and also drew attention to areas for improvement. The perspectives gathered may serve to guide other cross-sector collaborations across health and education to respond to OM among children at risk for OM-related disability in speech and language development.

**Electronic supplementary material:**

The online version of this article (10.1186/s12887-018-1081-3) contains supplementary material, which is available to authorized users.

## Background

Otitis media (OM) or middle ear infection is one of the most common childhood infections in children [1, 2] and a major public health burden even in developed countries [3]. A recent review estimated the prevalence of OM-related hearing impairment world-wide in under-fives to be 1 in 300, and that 21,000 die annually due to OM complications [4]. In Australia, urban and remote Aboriginal and Torres Strait Islander children experience OM at very high rates in comparison to non-Indigenous Australian children [5, 6]: in 2001 the prevalence of OM among 709 Aboriginal children aged 6–30 months across 29 remote communities from northern and central Australia was 91% (95% CI 88, 94), and perforated eardrums (an OM complication) affected 40% of children 0–18 months of age [7]. Overall, Aboriginal and Torres Strait Islander people comprise 2.8% of the Australian population, but more than a third (34%) of Aboriginal and Torres Strait Islander people are aged under 15 years, compared with 18.3% of the non-Indigenous population [8].

In Australia, the public health burden is particularly acute for Aboriginal and Torres Strait Islander children for whom OM tends to occur earlier in life (often by 8 weeks of age [9]), is more severe, and persists longer (cumulatively for 2.7 years on average, versus 3 months for non-Indigenous children [5, 10]). Among Aboriginal and Torres Strait Islander children, OM prevalence peaks at an early age: 72% at 5–9 months in Western Australia, according to a 2008 study [11].

Such chronic, severe OM at an early age is likely to have effects on speech and language development, hearing health, and later school readiness of a large number of Aboriginal and Torres Strait Islander children in Australia. Despite conflicting views on the impact of OM [12–16], it is clear that children growing up in poverty have higher risk factors for OM (e.g. poorer hygiene, housing, and second hand smoke) and that their speech and language development is especially at risk from OM [17, 18]. The ‘same’ experience of OM may more negatively affect a child growing up in adverse circumstances (crowded housing, poorer nutrition and immune function, lower income and lower education levels of parents, and/or living in remote area including access to medical specialists [19]). In terms of speech and language, OM clearly affects spatial hearing and receptive language skills [20]. Age is a likely factor: OM at 12–18 months is a known risk to speech and language development [16], and spoken language development has a cumulative, cascading development that begins prenatally due to auditory maturation. Within the first year of life, then, reduced auditory input through conductive hearing loss (fluid build-up in the middle ear and/or tympanic membrane perforation) is likely to delay and/or perturb auditory and neural attunement to native language speech sound categories, an early building block in spoken language development [13, 21] which is itself a foundation for school readiness. Long-term effects on spoken language, hearing health and school readiness are also possible through sensorineural hearing loss consequent to OM [22–26]. Further, recent animal research [27] suggests that more insidiously, the reduction in sensory experience due to OM-related conductive hearing loss may over time lead to degeneration of cochlear innervation and central functioning.

There are ongoing, intensive efforts to ‘close the gap’ in health outcomes between Indigenous and non-Indigenous Australians, but improvements in ear health and hearing are challenging. Indigenous infants are fitted with amplification at higher rates than previously, but only 2% of Indigenous infants provided with devices are fitted by 12 months compared with 10% for non-Indigenous infants (2013 data) [28]. Hearing health awareness campaigns have led to increased awareness of the signs of hearing loss but environmental conditions which predispose infants to OM continue to prevail particularly in remote communities, as previously noted (e.g. overcrowded housing, second-hand smoke and poor nutrition) [29]. Families are also in many cases anxious to avoid children being removed by government officials in child welfare, and diseases of poverty (e.g. scabies) can call attention to a family. OM is likely underdiagnosed in these circumstances. Medically, progress in treating OM remains slow, especially in remote areas, where there is generally a much higher burden of chronic disease and where access to specialist doctors is limited.

Public health efforts in hearing and ear health can potentially be more effective if they recognise existing crosscultural beliefs and practices. For example, Aboriginal parents may prioritise their children’s understanding and receptive language as opposed to their verbal performance [30]. There is also relatively little known about Aboriginal parents’ understandings of hearing health and to what extent they prioritise this. This is a particular issue since an auxiliary sign language is a common mode of communication with young children, including preverbal children [30].

By school age, many Indigenous children are regarded as developmentally vulnerable or delayed relative to non-Indigenous peers, for example on the Australian Early Development Census (AEDC [30]), a measure of teacher-rated school readiness across five key domains (including language and communication) administered in the first term of compulsory schooling (at age 5–6 years). In the Elsey NT region (2015 AEDC data), Indigenous children are between two and seven times more likely to be classified as “developmentally vulnerable” (<10th percentile in any domain) [31].

One local initiative to prevent disability associated with OM has been Learning to Talk, Talking to Learn (the LiTTLe Program). The LiTTLe Program was an early childhood language and hearing program which took as its starting point the reality that in remote Aboriginal communities many infants and children under 3 years of age have chronic OM. The LiTTLe Program was developed by general practitioner Dr. Fred McConnel and teacher of the deaf Robin McConnel and was in operation from 2006 until 2014 in remote communities east of Katherine, Northern Territory, through Sunrise Health Service, an Aboriginal controlled primary health service.

The LiTTLe program took a public health approach: it was open to all children 0–3 years, whether or not they were known to have OM. The program aimed to be preventive of poor long term speech and language outcomes in the home language. The logic of the program was that for children with chronic OM, who have fluctuating hearing loss, there are some times when hearing levels are relatively good. If speech and language input to children is intensified at such time windows, this should boost children’s speech and language development. Such increase in progress should buffer their speech and language development against the delays that might otherwise be expected due to OM. As it is not feasible in home or community settings to identify those windows of good hearing with any precision, the approach of the LiTTLe Program was to encourage parents and caregivers to increase the amount they talked with children in the 0–3 year age range, in home language, in a structured environment that promoted school readiness and support for the health issues of the child and their family.

The LiTTLe program approach was relatively intensive, and parent-implemented. Support was provided by other Aboriginal community members as everyday program workers (trainee early childhood teachers), and by a visiting manager to assist with programming, assessment and other administration. Both the visiting manager and the local community members listened to the concerns of parents and support with advice and referral to the community health clinic. The program was 4 h per day (i.e. half day attendance), 5 days per week in school terms (40 weeks per year). The program location was typically an underused school space, such as a spare room or verandah, with some activities run outside in more public areas. Program activities included parents talking with children while playing in a group setting (e.g. with blocks, picture books, outside with a garden hose), fun activities to support early literacy and familiarity with school type resources (e.g. painting), as well as school type breaks for morning tea, with health measures such as hand-washing and nose-blowing. The LiTTLe Program was free for families, and ran 2006–14, when the program was discontinued due to a reduction in Australian Government funding for Indigenous health. Funding was provided by: the Honda Foundation, Ian Thorpe Fountain for Youth, and the Federal Government’s Communities for Children Program (facilitated in the Katherine, Northern Territory, by The Smith Family, a non-government charitable organisation).

### Aim

In this study, the aim was to investigate the views about the LiTTLe Program among parents and other caregivers (such as grandparents, whom we henceforth refer to as “parents”). Our objectives were to find out about the speech and language strategies they were taught to use, what they thought about hearing and the promotion of hearing health in the program, and to what extent they considered that the LiTTLe Program assisted in their children’s school readiness.

## Methods

### Participants

Nine parents and caregivers (eight mothers and one grandmother) volunteered to participate in individual interviews. Participants were recruited by face-to-face approach from two members of the research team, one a local Aboriginal community member. All participants were provided with a formal participant information sheet and consent form, and gave informed consent in writing. Two prospective participants declined, citing work or family obligations. There was no apparent difference between those who participated and those who declined. All participating adults at no time had worked for the LiTTLe Program. All participants were assured confidentiality. Sampling was ongoing until any further participants were unavailable. Our research took place in one small Indigenous community in the Northern Territory and we worked to recruit as many parents as possible who had been in the LiTTLe Program. The population of the community (national census 2011) was 313, with 70 families of 2.2 children on average, 80% identifying as Aboriginal and 2.4% as Torres Strait Islander, and with 40% of the adult population in full-time work and 35% unemployed [32]. The 9 adult female participants represent more than half of the total number of mothers and caregivers (14) who participated in the program in this community over 2013–14. Given the small size of the community we estimate that the number of mothers with young children in the right age group for the program was about 15–20.

### Interview method

Our research team collaboratively developed a semi-structured qualitative interview. The input of university-based researchers and community-based researchers was iteratively incorporated. The wording was checked with the project’s senior Aboriginal mentor from the community and piloted to ensure clarity. The areas of focus were chosen based on the lead researchers’ observational experience of the LiTTLe Program while it ran (in 2013–14), knowledge of program goals, and experience in interviewing staff and other professional stakeholders for an industry report [33]. The interview began with open-ended questions in order to determine the overall views of parents while minimizing bias. Then the interview moved to ask parents their views about (1) speech and language strategies in the program, (2) hearing health and intervention, and (3) whether or how the program had improved school readiness in their children. The interview schedule is provided in Additional file 1.

Interviews were conducted over the period August to September 2015. This was 14 months after the LiTTLe Program concluded. Each potential interviewee first read a participant information statement and then signed an informed consent form, unless they preferred to listen to the information and give verbal consent instead (an option allowed by our ethics approval). All interviews were scheduled at a time and location of the participant’s choosing, e.g. outdoors at home, on school verandah, or in a spare office in the workplace. Children were not present during interviews. Interviews were conducted in English, in which all participants were highly proficient. Interviews were conducted on an individual basis to maximise the opportunity to hear more detailed views from each participant than has been our experience with focus groups in this context. Each interview was conducted by a local Indigenous community member together with a non-Indigenous academic researcher. Each interview was audio-recorded for later transcription and review. Participants had the option to decline to be audio recorded and one parent took this option. In that interview the participant’s responses were written down in note form during the interview.

### Data analysis

Each parent’s interview lasted approximately 45 min. The interviews were transcribed verbatim by a professional transcription service from the audio recordings (except one which was not audio recorded, at the parent’s request, and for which notes were taken during the interview). The information from parents was then grouped into five themes by one interviewer using NVivo (version 11.1.1): (1) overall views about the program, (2) views about speech and language strategies, (3) views about hearing health and intervention, (4) views about whether or how the program supported school readiness, and (5) views about program implementation and ideas for the future. Within these themes, responses were also grouped in a more fine-grained way into subthemes that emerged in our thematic analysis.

We also included some questions which listed options for parents to indicate, for example, via ranking how often certain foci had been promoted (on a scale of 1–3, “a bit”, “some”, or “a lot”). To analyse these results, the ranking level (1, 2, or 3) was multiplied by the number of adults who selected that option.

## Results

### Description of participants

Nine parents took part in interviews: eight mothers aged late teens through mid-twenties, five first-time mothers, most with one or two young children, and the grandmother in her mid-50s. Their children at the time of the program (as at April 2013) ranged in age from newborn to 4 years of age: one newborn, three 1-year olds, one 2-year old, and seven 3–4 year olds. The parents had grown up locally, except the grandmother. All parents reported using Kriol [34] as their main language at home, although English was also used in some families and is the language of schooling, and some parents spoke a little of their traditional language(s). All parents had participated in LiTTLe Program over 2013–14, four for 1–2 years, four for 2–3 school terms, and one for less than a school term. Some had attended every day, others 1–2 times per week. Each parent reported on one or more children’s experience in the program.

All parents had experience in managing OM in their children. The summary of medical ear histories in Table 1 documents early, chronic OM, complications and referrals, and relatively few with amplification. All children had been treated with medications such as amoxycillin.

**Table 1:** Medical ear histories of the children whose parents we interviewed

Measure	Data
Number of children who had had (bilateral) OM	12/12
Age at first OM episode	range = 2.0–12.8 months
	median = 5.0
Number of clinic visits at which OM was documented	range = 6–41 visits
	median = 15
Age at most recent OM episode	range = 32–87 months
	median = 64 months
Number of children with perforations of the tympanic membrane	6/12
Number of children referred to ENT (ear nose and throat specialist)/audiology	7/12
Number of children who had tried or used amplification	2/12

### Overall views

Parents had generally positive attitudes towards the program, with free responses ranging from “really good”, “good”, “interesting” to “all right”. The most positive responses were accompanied by comments emphasising the learning that the program had afforded for their children, for example to prepare them for school:

“Well, it was good. Kids who went, they learned, you know. They might have done better at preschool [than otherwise].”

“Really good, like, bringing [child’s name] in for more learning and all that, yeah.”

The parents who took positive views of the program also emphasised the fun the children had had, either when they were indoors drawing, painting, cutting out and listening to stories, or when they went outdoors to play. Parents who were more lukewarm about the program tended to report having participated in the program when there were fewer families and/or staff involved:

“It was all right. … Yeah, there were not many workers there.”

Parents tended to report that the good things about the LiTTLe Program included their child’s enjoyment of the activities. They tended to emphasise activities involving special resources: blocks, special ride-on toys, and school-type resources like scissors and glue, or activities outside. Two parents specifically mentioned oral interaction as good things about the program, for example:

“Doing things with kids. That was all right. Letting them talk and that.”

“No, it was good. Yeah, good all the way. When I brought her in every morning, she loved played, talking, yeah, having fun.”

Perceived downsides included physical resourcing, the programming, and the social aspects of group size. Some parents noted how toys had been broken over time, and there were in the end not enough toys to play with. One parent noted that the children were only given fruit and it would have been better to have more substantial food such as sandwiches. Some parents had wanted to go outside more often with the children. For some parents, there had also been too few other parents and children and so not enough variety in playmates and peers at the time they attended (e.g. only three or four other children).

The LiTTLe Program was parent-implemented, and this feature was enjoyed by all parents: spending time with their peers or friends, comparing notes about their children’s development, and in some cases having their child in the company and care of familiar relatives. All parents we interviewed were well aware that the program was intended to be inclusive, not just for children with clear problems. Specifically, they viewed the program as intended to promote school readiness, e.g.:

“It was good for, bringing kids to the school, to let them learn…. Yeah, so one day maybe when they are four or five they can go – so they can learn to go to Transition [first compulsory year of schooling].”

Other aims noted by one parent each were to socialise children with other children their own age, and to support adults to talk to children.

Parents came to the program for a variety of reasons. Some parents thought it would be fun and their child would learn new things. One family came because their child saw a LiTTLe Program craft activity outdoors and wanted to join in. Several parents reported simply being happy to get out of the house and have something to do:

“Well, I thought, like, you know, it was so boring at home, we didn’t do anything so I took my daughter to the school and yeah, it was fun.”

A few parents were a bit worried about their child’s development, or wanted opportunities for their child to learn and/or to socialise with others. In this community - which like many remote communities has relatively few opportunities for paid employment – a couple of parents joined in the hope of finding a job working in the program.

As shown in Table 2, the main program focus that the parents recalled seeing was supporting parents to talk to children in home language and to get ready for school. The next most salient focus was giving them information about hearing problems, helping parents support each other, and showing parents different ways to talk to children. In the rest of this paper we focus in on parents’ views on speech and language strategies, hearing health, and school readiness.

**Table 2:** Activities which parents reported they had seen in program, by number of parent reports

	a bit (1)	some (2)	a lot (3)	total ranked score
Support and show parents how to talk more to kids	1	4	3	18
Help kids / families get ready for school routines & activities	1	4	3	18
Support parents to talk to kids in home language	0	2	4	16
Give parents more information about hearing problems	1	3	3	15
Help parents support each other	0	3	3	15
Give parents ideas about different ways to talk to kids	1	3	2	13
Help connect/refer kids to the health service	3	2	1	10
Help parents whose kids had hearing problems	2	1	2	10
Support parents and kids to get ready for English	3	1	1	8

### Views about speech and language strategies

A core aim of the LiTTLe Program was to support parents in increasing the children’s access to language, via increasing the amount of oral language to which children were exposed. The use of traditional and other hand signs was also encouraged by the visiting program manager. The staff were also trained in modelling a variety of ways to talk to children. The question arises as to how much of this aim translated into practice in the program.

The main program message, to use a lot of everyday talk in naturalistic interaction with children, appeared to have been not as salient as other messages, such as social interaction in general. For example, parents readily recalled that they had explained right and wrong, and how to treat others well, and specific communicative routines like Twinkle, Twinkle Little Star, or Heads Shoulders Knees and Toes, or other games. A couple of parents recalled learning to talk to children while doing interactive activities like playdough or blocks:

Parent: Kind of, yeah.

Interviewer: Yeah. What kinds of things?

Parent: Just like talking to the kids more, you know, yeah.

Interviewer: Yeah, yep. So what kinds of activities were you doing when you did that? Can you remember?

Parent: Playing blocks. And parcels yes.

Several of the parents reported enjoying the experience of talking to their children a lot during the program sessions.

Parents reported that staff had showed them language practices and routines that might help their children’s school readiness, like book reading but also variety in directive language. Table 3 shows the strategies which parents remembered being recommended in the program, from a range of strategies advanced in the language development literature as facilitatory strategies, at least for Western children. (In Table 3, the frequency reported against each message is the number of parents in each case who perceived it as an emphasis in the program, so the maximum possible for each message is 9.)

**Table 3:** Perceived emphases and messages from staff in program

	Frequency
Talk about what you see together	8
Help them play with other kids and negotiate	8
Point to things and name them to the kids	7
Talk about what you’re doing (e.g. as you play blocks)	7
Get down to kids’ level, get close	6
Wait for kids to talk, then respond	6
Talk slowly	5
Singing, music, dance, clapping	5
Teach the kids ‘please’ and ‘thank you’	5
Check the kids understand e.g. ask them to bring you things	5
Ask them questions	5
Talk loudly	4
Get them to look at your face	3
Use baby talk to kids	3
Use sign language (finger talk)	2
Follow along with what the kid says, adding more words	2
Encourage the kids to ask questions	0

As shown in Table 3, the staff reportedly emphasised general oral language strategies, socialization practices, and auditory strategies. The general oral language strategies aimed to increase exposure to spoken language in the context of joint attention, naming, parallel talk and responsiveness (e.g. “Talk about what you see together”, “Point to things and name them”, “Talk about what you’re doing”, “Wait for children to talk then respond”). Socialization practices included teaching “please” and “thank you” or helping children negotiate. Auditory facilitation or stimulation strategies included speaking more loudly, more slowly, with face-to-face contact at child level, and using singing, dancing and clapping.

We were interested in what parents thought about these strategies given that some of them (e.g. direct, face-to-face child-directed talk) would be expected to be less culturally familiar to Aboriginal parents. Parents reported that they enjoyed doing singing, music, dancing and clapping. They also enjoyed asking questions or giving instructions to check the children’s understanding, waiting for children to reply. Some parents found it easy to name things to their children and talk about what they saw together. Some of the parents reported that their children enjoyed the extra spoken interaction but other parents didn’t report that it made any difference to their child.

Asking parents which strategies they found somewhat alien (“too hard or too weird”) turned up diverse responses. Two parents said that it felt strange to talk slowly, and one parent thought it felt stupid to follow along with the child’s topic, adding new words. One found it hard to use parallel talk i.e. to describe what they were doing while playing blocks, for example. Another thought that it would have been strange to encourage the child to ask questions. One parent disliked teaching their young child to say “please” and “thank you”. One parent particularly disliked being asked to use baby talk with her kids and didn’t feel comfortable using baby talk. One parent explained that she had shared some concerns about her child with LiTTLe Program staff. They had agreed that her child was not talking much at all, but commented that they had not seen the parent talking to her child either. They encouraged the parent to talk more to her child which she did. As a result, within a few weeks, her child was more verbal to the parent and the program staff.

### Views about hearing health and intervention

Another major focus of the LiTTLe Program was hearing health. The program aimed to increase parents’ knowledge about hearing loss as well as to help them access services and manage hearing problems in their children.

From the program, parents reported several activities to prevent hearing problems. They reported nose blowing, and that they had taken it on as a routine at home. Parents also identified the importance of taking children to the clinic to get their hearing checked. One parent mentioned tissue spears, i.e. rolled up tissues put into ear canal to soak up fluid (see [35]); this was a parent who had previously learned this before the LiTTLe Program for her child with a hearing problem. Another parent reported knowing how to administer ear drops supplied by the clinic. These reports are consistent with the picture of parents with extensive experience managing their children’s OM. A couple of parents explained that staff in the LiTTLe Program were crucial in alerting them to hearing loss in their children and supporting them to go to the clinic. Parents did not report physical exercise and deep breathing which were advocated in the program to promote healthy ears, nose and lungs.

A broader interest was parents’ attitudes and beliefs about hearing. After reflection parents gave us the following things as important for their children to be able to hear: people talking (e.g. relatives like cousins they play with, their parents, aunt or uncle, or other family members calling out to them), birds singing, and music including toys that play music, and the sounds of electronic games. Also viewed as important for children to hear, from the age of one and up, were dangerous oncoming animals and vehicles such as water buffaloes (which wander into the community streets from the adjoining bush), dogs running towards them, cars - including speeding cars - and trucks. It was reported that young children also needed to be able to hear their parents and grandparents warning them not to touch the stove or other dangerous things in the kitchen. One grandparent said how important it actually was for her own safety that her young granddaughter could hear the smoke alarm when it went off, when she as a grandmother couldn’t hear it: “She told me Nanna, come on, let’s go”. For older children, examples of sounds of the classroom that were regarded as important to hear were the teacher’s voice or music in the classroom.

**Fig. 1:**
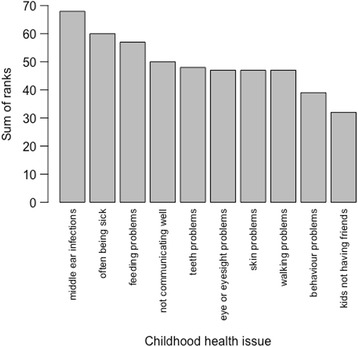
Ranked importance of health issues

We were also curious as to the possible relative importance of hearing loss for parents, given that many Aboriginal people in the region unfortunately live with multiple chronic health problems, including children from an early age. Parents ranked ten common issues in children’s health from most concerning to least concerning (i.e. which really matter and which they would want to do something about, down to those that can wait or are perhaps less important). This was not an easy exercise for parents. The results of the ranking across the 9 participants are shown in Fig. 1.

Somewhat unexpectedly, parents we interviewed ranked middle ear infections as their highest health concern in children aged 1–3 years. Other issues which parents ranked as major concerns in children of this age were ‘always being sick’ and feeding problems (i.e. nursing problems; mothers breastfeed infants in this community until they are two or three years old). At the other end of the scale, behaviour problems or children not having friends were openly explained by parents as not a concern for 1–3 year olds, who are expected to be somewhat autonomous and hard to manage, and not expected to have stable friendships yet. Consistently with their concern for OM, the parents were able to list several possible indicators of OM: child crying or cranky, pulling their ears, staring at people, and having a temperature and/or fluid coming out of nose or ears.

We were interested to follow up with parents about what in their view was appropriate intervention for infants and young children with a hearing loss. When we pursued the question of how to recognise a hearing problem in a very young child, however, such as a child of 6 months to one year, we received responses suggesting that our enquiries might have been perceived as overly focused on oral language communication. For instance, one parent said that 6-month-olds are too young to understand spoken language but do understand hand signs:

“They can understand us but it’s just that they are a bit too small to understand us. But they can do hand signs and things. And they can always like tell us that they need their lunches or water or fresh milk put in the bottle for them, or they can just drink it in a cup.”

With regards to the appropriateness of hearing aids for little children (e.g. 1–3 years old), parents reiterated that hand signs were a valuable way of communicating with young children with hearing loss. One parent said that using hand signs was a good option for children with hearing loss in remote communities rather than using speech:

Interviewee: Well, they can do hand sign to those kids. Yeah, talk to them.

Interviewer: So you think that’s better than hearing aids?

Interviewee: Yeah, it’s enough to just do hand signs.

One parent put hand sign in together with other strategies:

“It’s a good idea to use hand signs and for kids to use a hearing aid, and to keep checking their hearing.”

Most other parents reported that from what they had observed, hearing aids were not viable for very young children in the community because they tended to pull them out. There also seemed to be the perception among some parents at least that hearing aids were most important, that children were ready to benefit, only when they are three, four or five years of age, not when aged one or two. One 3-year-old child whose mother we interviewed had been happily trialling a hearing headband at the time of our interviews and the mother viewed this as appropriate.

### Views about school readiness

All parents thought that the LiTTLe Program had helped them get their child ready for school, in three main ways. First, parents reported that through the LiTTLe Program their child had learned social behaviours and cooperation required for school, as well as boosted their oral communication skills in general:

“Sharing, being nice when you play, caring, communicating”.

“Just learning, listening and getting involved with other kids, you know, communicating with other kids and that’s – I guess that’s how she felt confident to attend Preschool, yes”.

Second, parents reported that the school-type activities with similar resources were helpful in familiarising their child with early literacy and art activities. These included listening to stories, reading books with adults, counting, painting, drawing, colouring, finger painting, playing with a variety of toys, and learning to hold a pen. In addition, parents reported that through the LiTTLe Program their child became used to school routines and requests, such as how to get ready for school in the morning at home to have a shower and have breakfast in time to leave home, and how to stack books away at the end of an activity. These were things that one parent commented she would not have learned at home.

A third theme in the parents’ views on how the LiTTLe Program supported school readiness was in boosting their child’s confidence to move into formal schooling. One parent commented that her child “couldn’t wait” to attend Preschool.

In the LiTTLe Program, one intention had been to give the parents exposure to activities they could also use at home. All but one of the parents reported that they used some activities at home. These included practising writing the alphabet and numbers, playing with toys, playing with a homemade slippery slide, reading books, blowing their nose, painting and drawing, using their imagination to play office and play shop.

Another measure of school readiness might be in how the children settled in to school. We tried to ask this in the context of how the children compared with older siblings but many of the children didn’t have older siblings. It also turned out to be the case that after the LiTTLe Program some children went to creche before going on to Preschool. Some children settled in very well to school (“no problem”). Some experienced a week or so of crying at creche, some adjusted well from being initially quiet. Teachers told parents that their children were enjoying playing, numbers and books. One child took a year to settle in to school and still reacts badly to being in a large group in the classroom, one year on from being in the LiTTLe Program.

It was a decision by the LiTTLe Program to locate at the school so that children and parents would become familiar with the school grounds and with the routine of coming in to school. Many of the parents we interviewed thought it was good to have it at the school, and they appreciated the access to what they perceived as school resources like floor mats, paper, pens, kitchen, toilet, air conditioning, and a range of toys:

“Once she came in here it was good for her, she was so happy, she was happy to play in the school, playing with all the toys. She was happy to have a rest here and then every day after school I always went and picked her up. When she was two she came back and told me, “Mum, mum I play toys, I play toys”, she said like that and I said, “Oh this is the first time I’ve seen my daughter telling me that she was playing toys”.”

A few of the parents also reported to us that they thought that it was appropriate that an educational program was held at the school:

Interviewer: “That was a good place [school]?”

Interviewee: “Yeah.”

Interviewer: “Why was that a good place do you think?”

Interviewee: “For the kids to learn.”

One parent did report that their child got used to the school environment by attending the LiTTLe Program. They reported, however, along with some other parents we interviewed, that perhaps the location in the school was a turn-off to some of the parents who did not attend:

Interviewee: “She liked it, you know. And that’s how she got used to the school grounds. Yeah, because if it was somewhere else away from the school, I reckon she wouldn’t be comfortable coming to the school grounds and near the school. But yes, it’s a hard question.”

Interviewer: “A hard question, yeah.”

Interviewee: “Because yeah, maybe some would say no, it’s best for them to have it at another area.”

The other locations suggested by the parents were to hold the program near the shop or the Shire office, away from the school. They also suggested to hold it outdoors so that it would be easy for parents and children to look at it from a distance and see what was going on.

### Views about implementation and other suggestions

Parents who had had expectations about the program originally thought it would be bigger and cater for older children as well, like a creche. They did not expect (or regard it as) a health-oriented program, rather a generic early childhood program to support school readiness. Consistent with this, there were some reports of referrals to clinic for health issues, including children’s ears. But parents who had ongoing hearing problems themselves were very conscious of this health issue in any case, and said they took their children to the clinic for their ears anyway (and see high clinic visit rates, Table 1). Parents did not, however, report learning anything special about nutrition, despite it being a goal of the program to link with the clinic’s work in this area.

Regarding the engagement of the program with the community, several parents had recommended the program to a friend or relative e.g. other parents, friends, sister and cousin, but this wasn’t always successful. Parents tended to report three main reasons for non-attendance, as shown in Table 4: illness, family or community issues (disagreements, or needing to help older relatives), or travel (e.g. shopping, hospital, visiting relatives, ceremonies). Parents stopped attending altogether when bored with the range of activities, attracted by the full-day creche (which offered food), or when life became very complex.

**Table 4:** Reported reasons for non-attendance in program

	Frequency
Child was sick, or you were sick or you had to stay home and look after someone else	8
Family issues or community issues	6
Being away from the community for travel or ceremony	5
Transport	2
Not knowing if it was on	2
Needed childcare for an older child	1
Money issue	1

Parents suggested, that in future, several features would be desirable: more activities outside, more games and toys, a wider range of activities; a full day option including lunch; and more explanation about the program with the community.

## Discussion

This paper has explored the views of parents towards the LiTTLe Program, a program designed to prevent disability in terms of poor speech and language skills associated with OM in Indigenous children living in remote communities in northern Australia. We focused on the perspectives of Indigenous parents including their experiences, preferences and needs. These perspectives are not well documented in the peer-reviewed research literature on child disability and OM in Australia [36]. The program was intended to offer parents ways to access support for their children with OM within remote communities, and to build capacity within communities. This included, for instance, involving and upskilling community members, raising awareness of hearing within the school and clinic context, and supporting parents’ knowledge of ear health.

This paper has also documented the LiTTLe Program in terms of aims and approach. In itself this is a valuable contribution to the research literature in which interventions are underrepresented in comparison to the basic science of language development [37]. The LiTTLe Program differed from other current Australian programs to provide assistance to parents from disadvantaged and/or Indigenous families, the subject of a recent review [38]. LiTTLe differed from home visiting programs by involving parents in a social group setting, and differed from some programs that have a relatively narrow focus (e.g. on behaviour) by aiming holistically at improvements in language, health and school readiness.

Overall the study found that the LiTTLe Program had a generally positive reception among families, with some critical comments and insights as well. Below we discuss the main findings and their implications in speech and language strategies, hearing health and intervention, and then school readiness.

### Views on speech and language strategies

A significant area which the LiTTLe Program highlighted was the value of home language. The home language of many Indigenous infants and young to middle aged adults in this region of northern Australia is Kriol, an English-based creole. Kriol retains many aspects of traditional languages including a special ‘baby talk’ register which is widespread in traditional languages and contact languages (English-influenced varieties) of the region [30, 39].

Home life for young Indigenous people in this area is a mix of traditional and modern ways, and raising children is no exception. The comments from parents indicated a range of attitudes to baby talk, some less positive than documented so far in this region [30]. This may be a reflection of culturally less sensitive programs which promoted a middle-class non-Indigenous view of talking to children (sometimes negatively viewing baby talk, see [40]). It is also possible that our discussions about baby talk were really discussions about different kinds of registers relating to different child ages. We lack research evidence about how Kriol-speaking parents in this area modify their speech to infants under 18–24 months of age, for example, but research from other languages suggests that parents effectively adjust their speech to the abilities and level of the infant [41]. The parents did remark on the usefulness of conventionalised gestures for communicating with very young infants, and how this reduces the functional issue of hearing loss among children of this age, within this cultural context.

### Views about hearing health and intervention

Many of the parents and caregivers were quite knowledgeable about the physical and behavioural indicators of OM as it is prominently displayed in the clinic and in the community in public health posters, as well as having been a focus within the LiTTLe Program. Hearing health was clearly prioritised among parents in the context of other childhood health problems, as seen in the numbers of clinic visits for OM that we found. Some parents were also clearly sensitised to hearing loss because they had experienced it themselves.

What can be made of the finding that parents/caregivers viewed hearing aids as not workable or appropriate for young children in remote communities? It is a major concern to the hearing health provider (Australian Hearing) that Indigenous children tend to be fitted with a hearing aid around the age of 5 years, rather than within the first year of life. In this study, the participating parents provided us with an insight to the barriers to early fitting. It seems that at least in home contexts, hearing loss is not a major issue for communication since families routinely use sign language with young infants, a continuing cultural strength in this part of remote northern Australia [30]. Why are parents not opting for hearing aids for young children as they approach preschool age? The answer to this question is probably multilayered. Parents in the current study reported that young children in their community do not tolerate hearing aids well. This is consistent with other reports on limited usefulness of hearing aids in this pediatric population [42], which has high rates of supperative OM (non-infectious fluid in the middle ear) and where high levels of personal hygiene are hard to maintain.

Another factor is that Indigenous children also attend preschool at much lower rates than the non-Indigenous population [36], reducing the pressure to get ready for school environments and school interactions. Other factors which work against Indigenous parents seeking hearing aids for children are the risk of social stigma, an unwillingness to accept a disability label particularly when a health solution may not be a result, and fear of drawing attention to the family in the context of child removals (Stolen Generation as well as ongoing) and a newly increased level of government control over Indigenous people’s lives in Australia, especially in the Northern Territory following the Federal Government’s Intervention [43, 44].

To try to understand the perspectives of Indigenous families on the issue of hearing and children’s outcomes, and in part to see if there are other touchpoints that might be helpful from a hearing health provider’s perspective, we pursued a line of questioning which to our knowledge has not before been asked of remote Aboriginal families in Australia: for a young child, what is hearing useful for? Parents reported that it was important for children to be able to understand people such as relatives and other children, but also to hear environmental sounds particularly those which are cues to danger (e.g. cars, buffalo, smoke alarms), and to hear music and toys. Perhaps these insights about auditory information beyond speech are worth more focus in discussions between parents and hearing health providers about hearing aids.

Our findings that parents see issues with hearing aids for remote children has relevance for the use of sound field amplification systems in acoustically-treated classrooms. If children are less likely to have hearing aids, it is more imperative that a better hearing environment is created with rooms that damp echos and systems that provide the teacher’s voice clearly in the whole classroom. We observed in this part of the Northern Territory that some, but not all, schools use sound field systems. This highlights the role for collaboration and communication between health and education sectors, and the important role that teachers and education systems can play in reducing OM-related disability [36].

### Views on school readiness

Parents strongly valued the LiTTLe Program for school readiness, and they made several programmatic suggestions for improvement. These have practical implications for the LiTTLe Program but can also be seen as factors worth considering in other programs seeking to support remote Indigenous families. Parents resoundingly appreciated the school type resources and activities in preparing their children for school, and took the ideas home (an objective of the program). In the context of lower levels of preschool participation and less availability of quality preschool, the program was highly valued. Parents also engaged readily in demonstrations of early literacy strategies (e.g. shared book reading) and several alternative conversational strategies, even if they did not like them all. The strategies which parents regarded as alien or difficult -- talking slowly, following along with the child’s topic, using parallel talk, or baby talk -- suggest that some of the parents may be uncomfortable with a child-centred style of interaction. In a program like the LiTTLe Program there could be room for explanation of these strategies and considerable coaching of parents, or perhaps negotiation and reconsideration of the ways in which parents might want to adapt their speech and language to the child.

The location of the program was identified as possibly problematic by some parents, even as they appreciated that holding the program on school grounds was helpful in achieving school readiness goals. For many families, school does not hold many good memories. Additionally, school can still be perceived as a government domain (with links to the welfare system) rather than a place of cultural strength and positivity. Suggestions included more time outdoors in public areas which are visible and culturally safe. The program was very strongly associated in the parents’ minds with the community school because of its location, and viewed as an educational program rather than a combined health and education program. These points are feedback which may be instructive for other programs for disadvantaged and/or Indigenous families.

Many families in remote communities live in poverty and constant stress. Parents suggested running the program all day and with substantial food provision. The flipside of poverty and constant stress in a community with little paid employment can be boredom. Parents enjoyed the LiTTLe Program because of the opportunity to be with other same-aged adults in an unpressured group situation, which is naturalistic in remote communities, but wanted more social variety in peers for themselves and playmates for their children. Some parents joined the LiTTLe Program because it was “something to do”, or because they were actually looking for a job, or looking for help with a family or child situation. The LiTTLe Program offered a forum for families to build a relationship over time and seek help, as recommended in a recent review of Indigenous health [36].

Another significant outcome of the study is the recognition that there is a need for much greater cross-sector collaborations between health and education in Australia in relation to OM [36]. The LiTTLe Program offers a rare model of such a collaboration in responding to the high levels of OM and consequent disability among Indigenous children. While there are clearer recommendations in supporting school aged children, there are few options for early childhood programs [45]. The LiTTLe Program offers a model for reducing the gap within the early years of life, and has a potential to reduce the gap in health outcomes within a generation, meeting the goals of Australian Government’s Closing the Gap strategy [42, 46, 47].

### Limitations

This study was limited to 9 caregivers (8 parents and 1 grandparent) which is a small sample size even for qualitative in-depth interview work but similar to other work in small, remote, north Australian communities [48]. The sample size represents approximately half of the population of parent generation in the remote community, and the parents were an information-rich source as a key stakeholder group for the detailed understanding of this case [49]. This study explored caregivers’ perspectives on the LiTTLe Program among parents and grandparents who participated. It does not compare perspectives with caregivers who did not participate in the LiTTLe Program. In part this is because only caregivers who had participated would have detailed experience with the program. In part it would also have proved difficult to recruit caregivers to comment on a program they had not joined. Among the parents we interviewed, however, there were a range of attendance patterns and a range of attitudes. The caregivers were keen to provide feedback on the program, both positive and more critical. We tried to design our questions so as to capture both positive and negative feedback but (as a reviewer helpfully noted) we could have included the option ‘not at all’ (alongside ‘a bit’, ‘some’, ‘a lot’) in our questions regarding the extent to which parents saw certain foci promoted in the program.

## Conclusions

We identified generally positive attitudes to the LiTTLe Program among Indigenous caregivers in a remote community. The caregivers were mostly keen on the interactive strategies they were modelled in the program for supporting the children’s language and communication, and considered that the program very much helped them prepare their children for formal schooling. The caregivers also provided more critical perspectives on the approach of the LiTTLe Program, such as other options for location and services. These caregivers, who were experienced in managing OM and living with hearing loss, offered insights into the approach of the LiTTLe Program and the relevance of good hearing to their child and family.

The results of our study have relevance to community members and various agencies involved in Indigenous education and health. Childhood OM and hearing loss has long been recognised as a major public health problem among Indigenous children in Australia there have been relatively few coordinated efforts to address the resulting disability, across health and education. While there are relatively clear recommendations for supporting children with OM-related hearing loss once they reach school age, our findings offer clues towards approaches that may support children much earlier in life. While not perfect, there is much to be learned from the experience of the LiTTLe Program, especially from parents’ responses to this program.

## Additional file

Additional file 1: Interview schedule.This contains the full set of questions asked in the interview. For questions with other than an open response, it also contains the response options from which the participant chose. (DOCX 119 kb)

## Abbreviations

NT: Northern Territory (Australia)
OM: otitis media
OME: otitis media with effusion
