# Supplementary material for: A program to respond to otitis media in remote Australian Aboriginal communities: a qualitative investigation of parent perspectives
Source: BMC Pediatr. 2018 Mar 6;18:99. doi: 10.1186/s12887-018-1081-3 (PMC5840719; doi:10.1186/s12887-018-1081-3)
Supplement: Supplementary file 1 — Interview schedule.This contains the full set of questions asked in the interview. For questions with other than an open response, it also contains the response options from which the participant chose. (DOCX 119 kb) [file 12887_2018_1081_MOESM1_ESM.docx]

**LiTTLe Interview Questions**

These questions are asking your opinion:

1. What did you think about the LiTTLe Program, overall?

2. Was it different from a normal playgroup, or not? Tell me more.

3. What were the good things about the LiTTLe Program?

4. What wasn’t so good about the LiTTLe Program?

5. In your opinion, who was the LiTTLe Program for? (for some families or for everyone?)

6. Why did you join the LiTTLe Program?

7. In your opinion, what was the LiTTLe Program designed for? What were the staff trying to do?

8. Tick the box if you saw this going on in the program – a bit / some / a lot:

a bit some a lot

⬜ ⬜ ⬜ support and show parents how to talk more to kids

⬜ ⬜ ⬜ help kids / families get ready for school routines & activities

⬜ ⬜ ⬜ give parents ideas about different ways to talk to kids

⬜ ⬜ ⬜ give parents more information about hearing problems

⬜ ⬜ ⬜ help connect / refer kids to the health service

⬜ ⬜ ⬜ help parents whose kids had hearing problems

⬜ ⬜ ⬜ help parents support each other

⬜ ⬜ ⬜ support parents to talk to kids in home language

⬜ ⬜ ⬜ support parents and kids to get ready for English

9. Sometimes the staff were encouraging parents to talk more to children. Did you get involved in this? What did you learn to do? How?

10. How did this feel, talking a lot to kids? Did you like it or not? Why or why not? Did it make any difference to your child, compared with their older brothers/sisters?

11. Do you now find yourself talking more to your other younger kids?

12. What ways to talk to kids did the staff show you in the LiTTLe Program? Like during play on the mat or outside, or during activities like playdough, or reading books? Tell me more about that.

13. Tick the box if the staff were on about this idea a lot:

⬜ get down to kids’ level, get close

⬜ get them to look at your face

⬜ talk loudly

⬜ talk slowly

⬜ use sign language (finger talk)

⬜ wait for kids to talk, then respond

⬜ singing, music, dance, clapping

⬜ point to things and name them to the kids

⬜ talk about what you see together

⬜ talk about what you’re doing (e.g. as you play blocks)

⬜ help them play with other kids and negotiate

⬜ teach the kids ‘please’ and ‘thank you’

⬜ encourage the kids to ask questions

⬜ use baby talk to kids

⬜ follow along with what the kid says, adding more words

⬜ check the kids understand e.g. ask them to bring you things

⬜ ask them questions

14. Did you find it easy to try out any of the new ideas? Which ones?

15. Were any of the ideas really too hard or too weird? Did anything feel really strange or stupid so you didn’t want to do it?

16. We’ve got some cards here about health issues. We’d like to ask your opinion about which of these problems in a kid is most important to do something about. Can you put them in order so that the most important problem is at the top and the least important problem is at the bottom? Think about kids who are less than 3 years old.

17. Now, which of these do you believe hearing is related to? Put them together in one spot and put the other ones over here.

Can you tell me about these ones for hearing and why you put them there?

18. What is hearing important for, in this community or in your family? What is it important for kids to hear?

19. Did you learn anything in the LiTTLe Program in general about what a hearing problem looks like? (What do you see? How does the child behave? How can you tell, in a little baby at 6 months or 1 year old? What about how a kid is talking, can you tell from that? How do they tell you what they want? What do you have to do to get their attention?)

20. In the LiTTLe Program did you do any activities or learn about how to prevent hearing problems? What kinds of things? Do you still do any of those at home? Why or why not?

21. When you were in the LiTTLe Program did anyone suggest you take your child to the clinic for their hearing, or did you decide to go? Tell me more. How did that work out?

22. While you were in the LiTTLe Program, did your child ever need drops, ear spears, ear toilets, hospital visits, or ear operations? Did that work out? What was it like getting used to that? What did you have to do at home?

23. Did being in the LiTTLe Program change what you think about hearing problems?

24. What do you think about hearing aids for little kids (like 1-3 years old)? Do you have any experience with that or what do you think?

25. Did you think the LiTTLe Program helped you get the kids ready for school? (why or why not? in what ways?)

26. What new things did your child get to do that were supposed to get them ready for school?

27. What did you think about these things? (Were they useful or not? Did it seem to make any difference (compared with their older brothers/sisters)?)

28. How quickly did your kid settle in to school after the LiTTLe Program? (How about compared with other kids or their older brothers/sisters? What did the teachers think about how they settled in?)

29. Was it good to have the LiTTLe Program at the school or would it have been better in another location in the community? Did the location make a difference to your child, like how they talked or what toys they had?

30. What was it like being in the parent group?

31. Were there talking or school type activities from the program that you did at home? Are you still doing any of those activities? (Which ones?)

32. Did you ever recommend the program to a friend or relative?

33. Did the program change the way you think about how kids develop?

34. When you first went, what did you expect it would be like? Was it like that or not? How?

35. When you first went, why did you go? (How did you hear about it? Did someone recommend it; who – relative, friend, teacher, doctor? Was your child seeing anyone for a hearing or communication issue? Were you worried about your child’s hearing or communication? Was a friend of yours going?)

36. During the program did you end up going to the clinic to get any referral to ENT or specialist? (Did that work out? What happened?)

37. Did you get more information about nutrition or other programs from Sunrise through the LiTTLe Program?

38. On days when you didn’t go, why was that? Were there any bad times of year? Did the time of day work for you?

39. Did you ever have trouble going because of (tick what happened):

⬜ needed childcare for an older child

⬜ transport

⬜ money issue

⬜ being away from the community for travel or ceremony

⬜ not knowing if it was on

⬜ family issues or community issues

⬜ child was sick, or you were sick, or you had to stay home and look after someone else

any other reason?

40. Did you ever stop going? Can you tell us why (we won’t judge you, we just want to know)?

41. In the future, is there a way to make a program like this easier to attend, or more relevant or enjoyable? (how?)

42. How does your kid communicate now?

43. Describe a conversation with your kid now?

44. Do you talk with your kid about things that you did together in the past? Does your kid ever talk about that with you? Tell me more about that. Can you give me an example?

45. How well does your child listen and understand?

46. How well does your child get on with other kids?

47. Does your child have trouble hearing? Do they have a hearing aid? Do they have a referral or are they seeing an ENT?

48. What are your concerns now?
